# *TFF1* and* TFF3* mRNAs Are Higher in Blood from Breast Cancer Patients with Metastatic Disease than Those without

**DOI:** 10.1155/2018/4793498

**Published:** 2018-06-07

**Authors:** Marwa H. Elnagdy, Omar Farouk, Amal K. Seleem, Hoda A. Nada

**Affiliations:** ^1^Department of Medical Biochemistry, Faculty of Medicine, Mansoura University, Egypt; ^2^Department of Surgical Oncology, Faculty of Medicine, Mansoura University, Egypt

## Abstract

**Introduction:**

Breast cancer metastasis occurs when tumor cells dissociate from the primary tumor and migrate to distant organs through the peripheral bloodstream or lymphatic drainage. Circulating tumor cells (CTCs) originate from primary sites or metastases and circulate in the patients' bloodstream. Molecular assays for the detection and molecular characterization of CTCs can serve as a liquid biopsy and can represent an alternative to invasive biopsies as a source of tumor tissue in the metastatic patients.

**Patients and Methods:**

We analyzed the presence of CTCs in the peripheral blood of 50 breast cancer patients by quantitative real-time reverse transcriptase polymerase chain reaction (RT-qPCR) to detect* trefoil factor family *(*TFF*)* 1* and* 3* genes.

**Results:**

We found significant difference in the level of both* TFF1* and* TFF3* mRNA in the blood of nonmetastatic versus metastatic breast cancer patients (p**= **0.001 and p= 0.038, respectively).* TFF1 *mRNA was detected at higher levels in 34.6% of metastatic breast cancer patients as compared to 0% of nonmetastatic (p= 0.002). As regards* TFF3 *mRNA, it was detected at higher levels in 46.2% of metastatic breast cancer patients as compared to 4% of nonmetastatic (p= 0.026). Moreover, we found that the high level of both* TFF1* and* TFF3* mRNA was related to estrogen status of the patients. The detection of high level of* TFF1* mRNA in CTCs was associated with bone metastases (77.8%), while that of* TFF3 *was related to lymph node involvement (75%) and lung metastases (68.8%).

**Conclusion:**

The combined measurement of both* TFF1* and* TFF3 *mRNA level for differentiation of metastatic from nonmetastatic breast cancer gave 57.69% sensitivity and 83.3% specificity.

## 1. Introduction

Breast cancer is the most common malignancy and second cancer causing death in women. Breast cancer screening helps to reduce mortality by early diagnosis of cancer and metastases [1].

Metastasis is multiple steps process. It starts with the local invasion of surrounding host tissue by cells originating from the primary tumor. These cells then intravasate into blood or lymphatic vessels where they disseminate to distant organs. In the target organ, tumor cells adhere to capillary beds of this organ. Then, they extravasate into the organ parenchyma where they proliferate and start angiogenesis [2].

Circulating tumor cells (CTCs) are tumor cells that originate from primary sites or metastases and circulate in the patients' bloodstream. Early detection of breast cancer metastasis cannot be done by repetitive metastatic biopsies. So, the analysis of CTCs can provide an alternative liquid biopsy as easy and noninvasive method for diagnosis of metastasis [3].

Molecular assays for the detection and molecular characterization of CTCs are based on the isolation of total RNA from viable CTCs and subsequent qRT-PCR amplification of CTCs markers. This provides valuable information on the expression of cancer specific genes, or mutations of oncogenes and tumor suppressor genes, or epigenetic silencing of tumor suppressor genes and metastasis suppressors. This was previously done by the study of mRNA expression of a panel of marker genes on CTCs [4].

CTCs occur in blood at a very low concentration in comparison to millions of blood cells (nearly 1 CTC per 10^5^–10^8^ white blood cells) especially in patients with early-stage disease. So, CTCs should be firstly isolated and enriched before the characterization of genetic make-up using qRT-PCR [5].

Trefoil factor family (TFF) peptides are a family of growth factor-like peptides which was first proposed by Thim in 1988 [6]. They are characterized by the presence of unique three-loop structure stabilized by intrachain disulfide bonds in *α* 1-5, 2-4, 3-6 configuration between six conserved cysteine residues.

There are three TFF peptides: TFF1, TFF2, and TFF3. Their genes are clustered on chromosome 21q22.3 [7]. The expression of* TFF* genes has been detected in all tissues containing mucus-secreting cells, e.g., in gastrointestinal tract, ocular tissues, salivary glands, respiratory tissues, prostate, seminal plasma, cervical secretions, and milk. Their colocalization with mucus helps to form a stable gel-like mucus layer that plays a role in protection and healing of the mucosa from harmful agents, mechanical stress, viruses, and harmful pathogens [8].

TFF peptides are involved in mucosal maintenance and repair through motogenic and antiapoptotic properties. They also function as scatter factors, proinvasive, and angiogenic agents. So, they are important for digestive processes in the normal gastrointestinal tract where they act uniquely as tumor suppressors. In other organs, including the breast, their overexpression leads to cancer development and metastasis [9].

## 2. Patients and Methods

### 2.1. Blood Collection and Patients

Peripheral blood was collected into EDTA-tubes from 50 breast cancer female patients. They were classified into two groups: 24 cases of nonmetastatic breast cancer and 26 cases of metastatic breast cancer. They were treated in the Oncology Center, Mansoura University Hospital. Thirteen cases were premenopausal. Twenty-eight of the 50 patients had positive estrogen receptor in the primary tumor and 28 had positive progesterone receptor. The metastases were located in bone in 13 cases and in lung in 10 cases. Thirty-eight cases had lymph node involvement. Blood was taken from 14 healthy female volunteers. A written informed consent was taken before sample withdrawal. The study was performed in accordance with the ethical standards laid down in Mansoura Faculty of Medicine.

Peripheral blood mononuclear cells (PBMC) and CTCs were isolated from each blood sample by centrifugation through a Ficoll density gradient (Biocoll Separating Solution) with density 1.077 g/ml [10]. It was purchased from Biochrom-Gmb, cat. no. L 6113, Germany. Then, total RNA was then extracted using miRNeasy mini kit (Qiagen, cat no. 217004, Germany) [11]. RNA was quantified by spectrometry [12].

### 2.2. Reverse Transcription of Extracted RNA to Produce cDNA [13]

One *μ*g (1000 ng) of RNA was reverse-transcribed using Maxima® First Strand cDNA Synthesis Kit provided by Thermo Scientific, USA, cat. no. #K1641. The volume of RNA taken was calculated for each sample separately according to RNA concentration measured by nanodrop. The reaction was done by adding the following to calculated volume of RNA: 4 *μ*l 5X reaction mix (containing the remaining reaction components: reaction buffer, dNTPs, oligo (dT), and random hexamer primers) and 2 *μ*l maxima enzyme mix (containing maxima reverse transcriptase and Thermo Scientific Ribolock RNase inhibitor) and the reaction was completed to 20 *μ*l by nuclease-free water. Thus, each 1 *μ*l of the reaction contains 50 ng of RNA. The tubes were incubated for 10 minutes at 25°C followed by 15 minutes at 50°C. The reaction was terminated by heating at 85°C for 5 minutes.

PCR was done using the 2x PCR master-mix solution (i-Taq) provided by iNtRON Biotechnology to check for Tm and product length [14]. It was done in a total reaction volume of 20 *μ*l using 10 *μ*l PCR reaction mixture (1X), 1.6 *μ*l template DNA (80 ng), 0.8 *μ*l of 10 *μ*M forward primer (400 nM), 0.8 *μ*l of 10 *μ*M reverse primer (400nM), and 6.8 *μ*l distilled water.

Gene-specific primers were purchased from Invitrogen by Thermo Fisher Scientific. Primer sets for the PCR amplification genes were selected after testing the sequence of the three genes from NCBI database [15]. Then, these sequences were submitted in Primer3 tool and checked for product length, melting temperature, GC ratio, self-complementarity, and 3′ complementarity.

The following assays targeting specific mRNAs were included in the study: Homo sapiens* TFF1* mRNA (forward primer 5′- CCC-AGT-GTG-CAA-ATA-AGG-GC-3′ and reverse primer 5′- GCT-CTG-GGA-CTA-ATC-ACC-GT - 3′), Homo sapiens* TFF3* mRNA (forward primer 5′-TTT-TCT-GTC-CCT-TTG-CTC-CC– 3′ and reverse primer 5′- CCA-CGA-CGC-AGC-AGA-AAT-AA -3′), and Homo sapiens* ACTB* mRNA (forward primer 5′- GTG-GCC-GAG-GAC-TTT-GAT-TG –3′ and reverse primer 5′-GTG-GGG-TGG-CTT-TTA-GGA-TG –3′).

### 2.3. Quantitative PCR Analysis [16]

Real-time PCR was done for quantification of* TFF1* and* TFF3 *gene using SensiFAST SYBR® Lo-ROX (purchased from* Bioline, London, UK, catalog number: BIO-94005)*. For each reaction, the following was used: 10 *μ*l of SensiFAST SYBR Lo-ROX (1X), 0.8 *μ*l of 10 *μ*M forward primer (400nM), 0.8 *μ*l of 10 *μ*M reverse primer (400nM), and 1.6 *μ*l of template (80 ng), and each reaction was completed to reach a total volume of 20 *μ*l by nucleases free water (6.8 *μ*l).

Initial denaturation was done by heating for 1 min at 95°C followed by 40 cycles of denaturation at 95°C for 5 seconds and annealing /extension at 60°C for 30 seconds in 7500 Fast & 7500 Real-Time PCR System (Applied Biosystem, Themo Fisher Scientific, Life Technologies Corporation, USA). Melting curve analysis was done after amplification to confirm the specificity of the product and to exclude the presence of primer–dimers.

The relative gene expression analysis was done by Delta Delta cycle threshold (DDCT) method, and the average DCT of the healthy volunteers for each target gene was used as the calibrator sample [17]. The amount of target, normalized to an endogenous reference and relative to a calibrator, was calculated. The fold change is obtained by 2^–DDCT^. This method assigns a value of 0.7 to the calibrator sample, and all other quantities are expressed as an n-fold difference relative to the calibrator.

## 3. Statistical Analysis

Data was analyzed using SPSS 21. Parametric data were expressed in mean ± standard deviation. Nonparametric data were expressed in median, minimum, and maximum. Normality of data was first tested by one sample K-S test. Mann–Whitney was used to show the difference in the median value of* TFF1* and* TFF3* mRNA between nonmetastatic and metastatic groups. Fisher's exact test was used to show number and percentage of their high and low expression between studied groups. P value < 0.05 was considered as statistically significant.

## 4. Results

In our study, all cases were females. They were cross-matched with each other as regards age. The mean age of cases was 57.5 ± 7.6 years for nonmetastatic group and 56.0 ± 12.3 years for metastatic.

**Figure 1:**
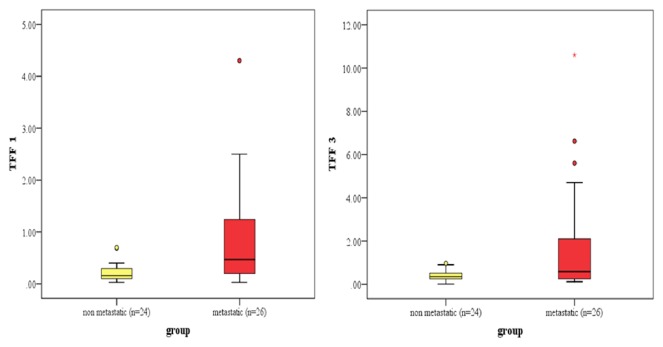
Box-whisker plot illustrates median (min.-max.) of* TFF1* and* TFF3 *mRNA level among the studied groups.

**Figure 2:**
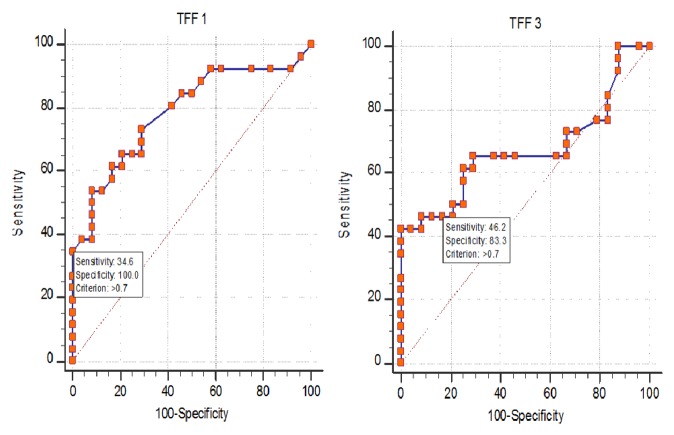
ROC curve illustrates performance analysis of* TFF1* and* TFF3* mRNA level within metastatic and nonmetastatic groups.

**Figure 3:**
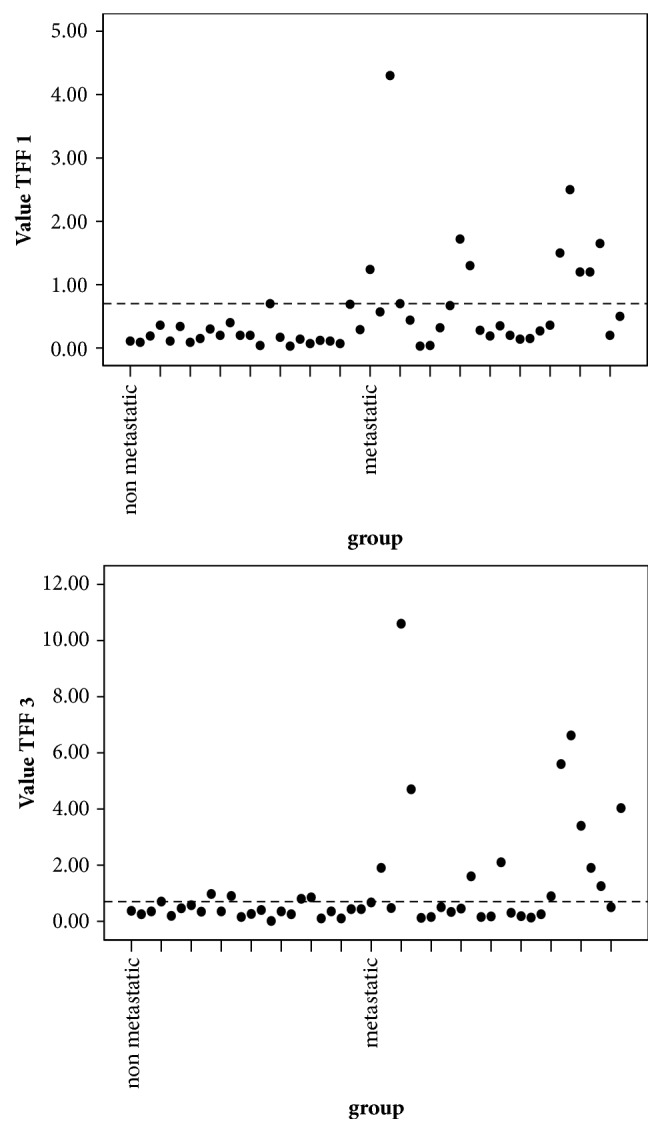
*Dot plot* chart illustrates distribution of high* TFF1* and* TFF3* mRNA levels within studied groups.

There was significant difference in the median value of* TFF1* and* TFF3* mRNA level between nonmetastatic and metastatic groups*** (Table 1, Figure 1).*** The value of 0.7 units was considered as cut-off value for both* TFF1 *and* TFF3* mRNA level, above which samples were considered to have higher concentration and below which samples were considered to have low concentration*** (Table 2, Figure 2).*** For both* TFF1 *and* TFF3*, this cut-off value was calculated by the median of the fold change of the calibrator samples calculated by 2^–DDCT^ method. According to this value, 34.6% of metastatic breast cancer patients showed high level of* TFF1* mRNA as compared to 0% of nonmetastatic. Regarding* TFF3* mRNA level, 46.2% of metastatic breast cancer patients had higher level as compared to 4% of nonmetastatic*** (Table 3, Figure 3).*** Cases with high* TFF1* mRNA were analyzed in relation to the estrogen status of patients to show that 88.9% were premenopausal*** (Table 4)***, 66.7% had ER positive primary tumor, and 44.4% had PR positive primary tumor. The analysis of patients with high* TFF3* mRNA concentrations revealed that 68.8% and 56.3% had ER and PR positive primary tumor, respectively*** (Table 4).***

The high level of* TFF1* mRNA was related to the site of metastasis to show that 77.8% of cases had bone metastasis, while 75% of cases with high* TFF3 *mRNA level showed lymph node involvement and 68.8% showed lung metastasis*** (Table 4)***.

## 5. Discussion

In this study, we measured the level of both* TFF1* and* TFF3* mRNA in nonmetastatic and metastatic breast cancer patients. This was done by measuring mRNA in RNA isolated from blood by density gradient centrifugation.

We found significant difference in the concentration of* TFF1* mRNA in the blood of nonmetastatic patients versus metastatic (p=0.001). The median was 0.16 (0.03-0.7) in nonmetastatic as compared to 0.47 (0.03-4.3) in metastatic patients. The median value of 0.7 units for* TFF1* mRNA detected in cells isolated from peripheral blood of fourteen healthy women was defined as the threshold below which samples were considered to have lower* TFF1 *mRNA concentrations and above which samples were considered to have higher concentrations. This was used as the cut-off value to differentiate nonmetastatic from metastatic patients. This value gives us 34.62% sensitivity and 100% specificity.

In the present study, the level of* TFF1* mRNA was different significantly between the 2 groups (p=0.002). It was high in 34.6% of metastatic breast cancer patients as compared to 0% of nonmetastatic. This is in agreement with the results described by* Lasa et al.* [4] which showed that* TFF1* is positively expressed in the blood of 17% of metastatic breast cancer patients.

As regards TFF3, we found significant difference in the level of mRNA in the blood of nonmetastatic versus metastatic patients (p=0.038). The median of* TFF3 *was 0.35 (0.01-0.97) in nonmetastatic as compared to 0.585 (0.12-10.6) in metastatic group. The median value of 0.7 units for* TFF3* mRNA detected in cells isolated from peripheral blood of fourteen healthy women was defined as the threshold below which samples were considered to have lower* TFF3* mRNA concentrations and above which samples were considered to have higher concentrations. This value gives us 46.15% sensitivity and 83.33% specificity.

In our study, there was significant difference in the level of* TFF3 *mRNA between the 2 groups (p=0.026). It was high in 46.2% of metastatic breast cancer patients as compared to 4% of nonmetastatic. This is in agreement with the results of study done by* Livak and Schmittgen *which showed that* TFF3* is positively expressed in the blood of 20% of metastatic breast cancer patients [17].

The higher levels of* TFF1* and* TFF3 mRNA *in CTCs of some metastatic breast cancer patients confirmed that they act as tumor progression factors. This might be explained by the role described by* Chaiyarit et al. *as signal transducers to decrease apoptosis, increase tumor cell motility, and increase angiogenesis [18].

On the other hand, the low level of* TFF1* and* TFF3* mRNA in the remaining metastatic breast cancer patients may be consistent with the results of* Buache et al.* [19] who proposed that the TFF1 and TFF3 play a role during mammary gland morphogenesis, ontogenesis, and remodeling. So, loss of these functions can lead to cancer progression [20].

Among the 9 cases of metastatic breast cancer (MBC) that have high* TFF1* mRNA level, we found that 8 cases were premenopausal (88.9%). This may confirm the results of the retrospective study of* Markićević et al.* [21] which showed that levels of TFF1 expression were significantly higher in breast tissue samples in premenopausal patients than in postmenopausal (p=0.02). The results of* Ishibashi et al.* [22] showed that serum TFF1 in breast cancer patients who were immunohistologically positive for TFF1 was significantly higher than patients who were immunohistologically negative for TFF1 (P= 0.017). Also,* Bohn et al.* [23] noticed in their study that there was no significant difference between MBC and primary breast cancer (PBC) groups (P > 0.05) as regards expression rates of TFF1.

Within the 9 cases of MBC that have high* TFF1* mRNA level, we found 6 cases with ER + primary tumor (66.7%). This is in accordance with the results of* Haakensen et al.* [24] and* Markićević et al.* [22] who showed that TFF1 was differentially expressed according to serum estradiol levels and it was higher in patients with ER+ breast cancer.

The previous results were in agreement with the study done by* Prest et al. *which concluded the presence of promoter containing an estrogen-response element (ERE) that regulates the expression of the TFF1. The induction of TFF1 is a primary response to estrogen and it is mediated by the binding of the estrogen receptor complex to a 13-bp near-palindromic estrogen-response element, GG TC AC GG TG GC C, located 400 bases upstream of the* TFF1* transcription start site [25].

As regards* TFF1* mRNA concentration in serum samples of PR + breast cancer patients, we found that only 4 cases with high* TFF1* were PR+ (44.4%). These results were different from the results of Markićević et al. [21] who showed that* TFF1* was higher in patients with PR + breast cancer. They explained this by the fact that progesterone is estrogen-regulated protein and its level is related to estrogen level.* Crosier et al.* [26] proved that there was a relationship between the expression of the estrogen receptor, progesterone receptor, and TFF1 in breast cancer. In our study, the small sample size may be the cause of this difference.

As regards the level of* TFF3* mRNA in CTCs in ER + breast cancer tissue, we found 68.8% (11 of 16) of patients with high* TFF3* level had ER + primary tumor. This may confirm the results of* Ahmed et al.* [27] who stated that TFF3 protein expression is associated with estrogen receptor expression. They found that TFF3 expression was not detected in most of the tumors that do not express the estrogen receptor. There was a strong positive correlation between estrogen receptor and TFF3 protein expression in tumor tissue. They found also that the levels of TFF3 expression in the primary tumor were associated strongly with its level in the corresponding metastatic cells and the level was increased as the tumor cells moved along the metastatic cascade from the primary tumor.

Among the 16 cases that had high concentration of* TFF3* mRNA, we found 9 cases with positive PR expression in primary tumor (56.3%). This is in accordance with* Ahmed et al.* [27] who had foundstrong positive correlation between progesterone receptor and TFF3 protein expression in tumor tissue.

The relation between the concentration of* TFF3* mRNA and estrogen status is explained by the results of* May and Westley* who stated that TFF3 is an estrogen-responsive gene, and its expression level is positively correlated with ER status in breast cancer.* Pandey et al.* [28] found that the coexpression of TFF3 and ER positivity in breast cancer increased tumor invasion and metastatic seeding.

We should focus on the fact that ER, TFF1, and TFF3 are expressed in normal breast epithelial cells and are important for the normal physiology of the breast epithelium [29]. All of them are under the control of estrogen. The switch from a beneficial to a malignant behavior may result from the loss of tissue architecture and matrix remodeling which posits the epithelial cells near fibroblasts, muscle, nerve, and endothelial cells in an invasive tumor in contrast to the compartmentalization provided by the myoepithelial cells in normal mammary gland. Besides breakdown in tissue architecture, the inversion of cell polarity facilitates direct secretion of TFF peptides into the ectopic location of the tumor stroma where it will exert its biological effects [27].

In our study, of the 9 cases of MBC patients that have high level of* TFF1 *mRNA, there were 7 cases with bone metastasis (77.8%). This is in agreement with the study of* Wang et al.* [30] who found that 43.3% of patients with bone metastasis exhibited a high expression level of TFF1. Similarly,* Smid et al.* [31] considered TFF1 most differentially expressed gene (P < .0015) in breast cancer metastasis to bone.

The fact that TFF1 may contribute to tumor metastasis to bone is proved by its high expression in MBC.* Emami et al.* explained this by the ability of TFF1 to dimerize with cysteine-rich molecules, as cysteine-rich intestinal protein 1 (CRIP1) which also may be overexpressed in breast cancer and might be an interacting partner for TFF1 [32]. The presence of ER positive primary tumors has the strongest association with metastasis to the bone [31].

In the present study, out of the 16 cases that had high* TFF3* mRNA concentration, we found 12 cases (75%) had lymph node metastases. This confirmed the observation of* Ahmed et al.* [27] that there was significant correlation between the overexpressed TFF3 in breast cancer and the lymph node metastases. They also found that the expression of TFF3 was higher in malignant cells that have metastasized away from than in those within the primary tumor.

In our study, we found 11 cases of high* TFF3* mRNA level had lung metastasis (68.8%). This is consistent with the study of* Pandey et al.* [28] who made forced expression of TFF3 in mice and examined their ability to form metastatic nodules. They found 4 of 6 mice with expressed TFF3 developed lung nodules. They explained this by the increase in hypoxanthine-guanine phosphoribosyl transferase (hHPRT) activity in lung of mice with ER+ mammary carcinoma cell lines that developed increased expressed TFF3.

Finally, we analyzed the results of the combined analysis of* TFF1 *and* TFF3* in the blood of breast cancer patients. They gave 57.69% sensitivity and 83.3% specificity. This means that if both genes are overexpressed in blood of breast cancer patients, the risk of development of metastasis will be 57.69%.

The use of* TFF1* and* TFF3* as markers in the CTCs of breast cancer patients is hopeful. Their upregulation is associated with breast cancer tumorigenesis. Their detection in CTCs of breast cancer patients confirms that the CTCs originated from the breast and maintains the properties of breast cancer cells [33].

The high concentration of* TFF1 *and* TFF3* mRNAs in women with ER + primary tumors is exciting. This may raise the possibility that the measurement of* TFF1 *and* TFF3 *mRNAs in cells isolated from peripheral blood can help the prediction of endocrine response, degree, and duration of response. The use of antiestrogens can antagonize estrogen mediated induction of* TFF1* and* TFF3* mRNA expression.

## Figures and Tables

**Table 1:** The median value of TFF1 and TFF3 between nonmetastatic and metastatic group.

	**Nonmetastatic group** **(n=24)**	**Metastatic group** **(n=26)**	**P value** **∗**
***TFF1*** **∗** Median(min-max)	0.16 (0.03-0.7)	0.47 (0.03-4.3)	0.001

***TFF3*** **∗** Median (min-max)	0.35 (0.01-0.97)	0.585 (0.12-10.6)	0.038

Significant P < 0.05; ^*∗*^Mann–Whitney to show the difference in the median value of *TFF1 *and *TFF3 *mRNA between nonmetastatic and metastatic groups.

**Table 2:** The performance analysis of *TFF1* and *TFF3 *mRNA within metastatic and nonmetastatic groupsconsidered the value of 0.7 units as cut-off for both *TFF*1 mRNA and *TFF3 *mRNA above which samples were considered to have higher concentration and below which samples were considered to have low concentration.

**Variables**	**AUC**	**Cut-off **	**Sensitivity** **(95%CI)**	**Specificity** **(95%CI)**	**PPV** **(95%CI)**	**NPV** **(95%CI)**
***TFF1***	0.784	> 0.7	34.62 (17.2-55.7)	100.0 (85.8-100.0)	100.0 (66.4-100.0)	58.5 (42.1-73.7)

***TFF3***	0.671	> 0.7	46.15 (26.6-66.6)	83.33 (62.6-95.3)	75.0 (47.6-92.7)	58.8 (40.7-75.4)

**Combined *TFF1*&*TFF3***	0.705	-------	57.69 (36.9-76.6)	83.3 (62.6-95.3)	78.9 (54.4-93.9)	64.5 (45.4-80.8)

**AUC:** area under the curve, **PPV:** positive predictive value, **NPV:** negative predictive value, and **CI:** confidence interval.

**Table 3:** The number and percentage of high and low levels of *TFF1* and *TFF3* mRNA between non metastatic and metastatic groups (regarding cut-off 0.70).

	Nonmetastatic group (n=24) No. (%)	Metastatic group (n=26) No.(%)	*p*-value
***TFF1*** *∗*			

High level	0 (0.0)	9 (34.6%)	**0.002**
Low level	24 (100.0%)	17 (65.4%)	

***TFF3*** *∗*			

High level	4 (16.7%)	12 (46.2%)	**0.026**
Low level	20(83.3)	14(53.8)	

Significant P < 0.05; *∗*Fisher's exact test to show number and percentage of their high and low expression between studied groups.

**Table 4:** The number and percentage of high *TFF1* and *TFF3* mRNA level in ER and PR positive and negative breast cancer patients. The number and percentage of high *TFF1* mRNA level in pre- and postmenopausal breast cancer patients, in cases with and without bone metastasis, and the number and percentage of high *TFF3* mRNA level in patients with and without lymph node involvement and lung metastasis.

	**High *TFF1* mRNA level (n=9)**		**High *TFF3* mRNA level (n=16)**	
	No.	%	No.	%
**ER**				

Positive	6	66.7	11	68.8

Negative	3	33.3	5	31.3

**PR**				

Positive	4	44.4	9	56.3

Negative	5	55.6	7	43.8

**Lymph node involvement**				

Present			12	75.0

Absent			4	25.0

**Lung metastasis**				

Present			11	68.8

Absent			5	31.3

**Menopause status**				

Premenopausal	8	88.9

Postmenopausal	1	11.1		

**Bone metastasis**				

Present	7	77.8		

Absent	2	22.2
